# Tackling Negative Symptoms of Schizophrenia with Memantine

**DOI:** 10.1155/2014/384783

**Published:** 2014-04-10

**Authors:** Antonios Paraschakis

**Affiliations:** Department of General Adult Psychiatry, Psychiatric Hospital of Attica “Dafni”, 16674 Athens, Greece

## Abstract

We present a case of a 52-year-old male patient suffering from chronic schizophrenia stabilized on risperidone long-acting injection (37,5 mg/2 weeks) and biperiden 4 mg/day. Residual symptoms are affective flattening, alogia, avolition, and asociality. Memantine 10 mg/day was added. After 1.5 months, the patient spontaneously referred to “feel better being in company of my relatives.” The following scales have been completed: the Scale for the Assessment of Negative Symptoms (96), the Scale for the Assessment of Positive Symptoms (3), the Mini Mental Scale Examination (26), and the Calgary Depression for Schizophrenia Scale (2). Memantine was increased to 20 mg/day and biperiden was decreased to 2 mg/day. Two months later, apathy and asociality considerably improved and affective flattening, alogia, and attention slightly got better (SANS 76, SAPS 1, MMSE 26, and CDSS 1). After two more months, the improvement continued in the same domains (SANS: 70, SAPS: 1 MMSE: 27, and CDSS: 1). Positive symptoms remained in full remission. It has been hypothesized that one of the causes of schizophrenia is glutamate excitotoxicity. Memantine, a glutamate receptor antagonist, could possibly ameliorate schizophrenia symptoms, the negative ones among them, used as add-on therapy to atypical antipsychotics. Memantine could be of potential help in schizophrenia patients with severe residual negative symptoms.

## 1. Introduction

Negative symptoms are a mainstay of chronic schizophrenia and constitute a cause of severe disability for the patients. The etiology of negative symptoms is complex; they could be due to the disease itself, secondary to positive symptoms, or due to medication's side effects; additional causes are depression and institutionalization [1].

Negative symptoms are resistant to the current pharmacological treatments. Even after the discovery of the novel or “atypical” antipsychotics, negative symptoms remain mostly refractory to treatment. Various medications have been tried as add-on therapies to atypical antipsychotics with modest benefit, at best: antidepressants, cholinesterase inhibitors, selegiline, *Ginkgo biloba*, modafinil, and armodafinil [1]. 

A recent research hypothesis regarding the etiology of schizophrenia suggests that one of its main causes is glutamate excitotoxicity; as a consequence, glutamatergic antagonists could hypothetically not only provide symptom relief but also be disease-modifying [2, 3]. Among the glutamate antagonists, memantine—a drug used in modest to severe Alzheimer's disease [4]—has been tried as an adjunct medication.

## 2. Case Presentation

We report a case of a 52-year-old male patient suffering from schizophrenia since the age of 22. He was receiving risperidone long-acting injection 37.5 mg every 2 weeks and biperiden 4 mg/day (due to extrapyramidal tremor). His prominent symptoms were the negative ones: avolition, apathy, asociality, affective flattening, and poverty of speech. 

The patient was stabilized on this treatment for 2 years and both he and the psychiatrist were very reluctant in switching antipsychotic. Risperidone very successfully dealt with the previously present positive symptoms (aggression, disorganized behavior, and persecutory delusions).

Aiming to tackle the patient's negative symptoms memantine 10 mg/day was added (memantine's use was off-label). One and a half months later, the patient spontaneously referred a change in his daily routine (“I feel better when I am in company of my relatives”). At that point a battery of psychometric tests has been completed: the Scale for the Assessment of Negative Symptoms (SANS), the Scale for the Assessment of Positive Symptoms (SAPS), the Mini-Mental State Examination (MMSE), and the Calgary Depression for Schizophrenia Scale (CDSS). The results were SANS 96, SAPS 3, MMSE 26, and CDSS 2.

Memantine was increased to 20 mg/day, maximum dose indicated in Alzheimer's dementia, and biperiden was decreased to 2 mg/day in order to facilitate the former's action. After 2 months, a considerable improvement was seen for the negative symptoms: SANS 76, SAPS 1, MMSE 26, and CDSS 1. The improvement was most pronounced for avolition-apathy (4 items in the SANS, −6) and anhedonia-asociality (5 items, −5); affective flattening (8 items, −6), alogia (5 items, −1), and attention (3 items, −2) slightly improved. The positive symptoms were practically nonexistent but they were almost absent even before memantine was commenced. Mild extrapyramidal tremor was tolerable by the patient; he agreed to biperiden being kept at 2 mg/day.

Two more months later, the patient continued to improve albeit in a less significant way: SANS 70, SAPS 1, MMSE 27, and CDSS 1. Improvement was seen for avolition-apathy (−2) and anhedonia-asociality (−2). The remaining domains showed minimal changes: affective flattening (−1), alogia (0), and attention (−1).

In particular, the patient's grooming and personal care as well as the relationship with his relatives were considerably ameliorated during all these months.

Memantine did not cause any additional side effects to the patient.

## 3. Discussion

Glutamate is the main excitatory neurotransmitter in the central nervous system [5]. According to a current research hypothesis, the glutamatergic system and specifically the N-methyl-D-aspartate (NMDA) receptors are hypofunctional in schizophrenia [6]. It is possible that the hypofunctional NMDA receptors could lead to a compensatory excessive glutamate release trying to overcome that deficit; reversing this trend may be helpful in reducing schizophrenia symptoms [7]. Furthermore, NMDA-receptor hypofunctioning could diminish central gamma-aminobutyric acid (GABA) tone and lead to a disproportionate release of glutamate into the synapse; this could result in extensive neuronal death [8].

Memantine is an NMDA-receptor antagonist that partially blocks NMDA receptors thus preventing a toxic influx of calcium and the resultant cell death [9]. It has been hypothesized that it could ameliorate schizophrenia symptoms—the negative ones among them [10].

Memantine has shown to improve agitation and delusions in patients with Alzheimer's dementia [11]. Furthermore, it does not seem to worsen the positive symptoms of patients with schizophrenia [6]. Therefore, memantine could possibly help in maintaining—and, in any case, not worsening—the improvement of positive symptoms achieved with the antipsychotic therapy. The slight improvement observed in the SAPS in our case probably confirms this action.

Furthermore, when serotonin binds to the serotonin 5HT-2A receptors, it accelerates glutamate release [12]. Therefore, atypical antipsychotics, which are also serotonin 5HT-2A antagonists, like risperidone, have the property of reducing toxic glutamate hyperactivity via serotonin 5HT-2A antagonism and, in turn, reducing mesolimbic dopamine release [12]. It comes up that the combination of memantine with a serotonin-dopamine antagonist could provide a synergistic effect.

The findings regarding memantine's adjunct to atypical antipsychotics in patients with chronic schizophrenia and residual negative symptoms are conflicting in the literature. Some authors report benefit [13–17], while others either refer no significant effect [6, 10] or that the data is inconclusive [18, 19]. However, memantine had been given at the maximum dose for only 9 weeks in the study by Lee et al. [6] and 6 weeks in the study by Lieberman et al. [10]. The patient in our case received memantine in full dose for 16 weeks. Furthermore, his negative symptoms were severe and, as Lieberman suggested, “memantine could be potentially helpful in patients with severe residual psychopathology or pronounced cognitive impairment” [10]. The view regarding its possible usefulness in cases of schizophrenia with cognitive impairment is supported by another study too [20].

Rezaei et al. [16] in their study used the same combination of medications as we did—risperidone + memantine—for tackling the negative symptoms in their sample. The authors suggested that, in order to evaluate improvement in primary negative symptoms, it is essential that the patient should be depression-free and without positive or extrapyramidal symptoms. Our patient fulfilled the first 2 criteria but not the third one (in fact, as will be mentioned later, this is a limitation of our study). It is possible that memantine could be a good choice for patients with schizophrenia sharing these characteristics.

A limitation of our study is that the patient was receiving an anticholinergic medication, biperiden, due to extrapyramidal symptoms. Anticholinergics are considered cognitive depressants and have been accused for worsening negative symptoms too [21]. Consequently, a part of the improvement seen could be due to biperiden's decrease. Another limitation is the fact that the psychiatric scales were not completed prior to memantine's intake; the remarkable initial response could have not been anticipated.

We conclude that memantine given in full dose for at least 4 months helped in improving specific negative symptoms in a case of chronic schizophrenia with well-controlled positive symptoms and no depression. The combination with a serotonin-dopamine antagonist—risperidone in our case—provides pharmacological benefits; it also proved well—tolerated. Memantine used as an adjunct medication in cases of schizophrenia with the aforementioned characteristics could be proposed as a worthwhile and safe augmenting option.
